# Evidence of How Physicians and Their Patients Adopt mHealth Apps in Germany: Exploratory Qualitative Study

**DOI:** 10.2196/48345

**Published:** 2024-01-17

**Authors:** Tanja Schroeder, Maximilian Haug, Andrew Georgiou, Karla Seaman, Heiko Gewald

**Affiliations:** 1 Centre for Health Systems and Safety Research Australian Institute of Health Innovation Macquarie University Sydney Australia; 2 Institute for Digital Innovation Faculty of Information Management Neu-Ulm University of Applied Sciences Neu-Ulm Germany

**Keywords:** mobile health apps, DiGA, adoption, prescription, mHealth, aging and individual differences

## Abstract

**Background:**

The enactment of the “Act to Improve Healthcare Provision through Digitalisation and Innovation ” (Digital Healthcare Act; *Digitale-Versorgung-Gesetz* [DVG]) in Germany has introduced a paradigm shift in medical practice, allowing physicians to prescribe mobile health (mHealth) apps alongside traditional medications. This transformation imposes a dual responsibility on physicians to acquaint themselves with qualifying apps and align them with patient diagnoses, while requiring patients to adhere to the prescribed app use, similar to pharmaceutical adherence. This transition, particularly challenging for older generations who are less skilled with technology, underscores a significant evolution in Germany’s medical landscape.

**Objective:**

This study aims to investigate physicians’ responses to this novel treatment option, their strategies for adapting to this form of prescription, and the willingness of patients to adhere to prescribed mHealth apps.

**Methods:**

Using an exploratory qualitative study design, we conducted semistructured interviews with 28 physicians and 30 potential patients aged 50 years and older from August 2020 to June 2021.

**Results:**

The findings reveal several factors influencing the adoption of mHealth apps, prompting a nuanced understanding of adoption research. Notably, both physicians and patients demonstrated a lack of information regarding mHealth apps and their positive health impacts, contributing to a deficiency in trust. Physicians’ self-perceived digital competence and their evaluation of patients’ digital proficiency emerge as pivotal factors influencing the prescription of mHealth apps.

**Conclusions:**

Our study provides comprehensive insights into the prescription process and the fundamental factors shaping the adoption of mHealth apps in Germany. The identified information gaps on both the physicians’ and patients’ sides contribute to a trust deficit and hindered digital competence. This research advances the understanding of adoption dynamics regarding digital health technologies and highlights crucial considerations for the successful integration of digital health apps into medical practice.

## Introduction

Mobile devices have enormous potential to enhance the way patients receive medical care and health education [1]. Mobile health (mHealth) is a dynamic and expanding area of health care with short innovation cycles [1,2]. mHealth is closely related to telemedicine and eHealth. The fundamental difference lies in how the related services are delivered to the patient. According to the World Health Organization (WHO), mHealth expands the spectrum of functionalities to include a mobile component. Thus, mHealth is delivered through any mobile device, from simple mobile phones, smartphones, and tablets to wearable devices used in health care settings [2].

To accommodate the growing number of mHealth apps (ie, software products for smartphones designed to support good health), Germany passed the “Act to Improve Healthcare Provision through Digitalisation and Innovation” (Digital Healthcare Act; *Digitale-Versorgung-Gesetz* [DVG]) in December 2019 and the Digital Health Applications Ordinance (*Digitale-Gesundheitsanwendungen-Verordnung* [DiGAV]) in October 2020 [3,4]. This ordinance enables physicians to prescribe mHealth apps (in German referred to as *Digitale Gesundheitsanwendungen* [DiGAs; digital health applications]) to their patients in the same way as any other medicine. A DiGA supports the recognition, monitoring, treatment, and alleviation of diseases, injuries, or disabilities [5]. To qualify as a DiGA, an mHealth app has to successfully pass the evaluation of the Federal Institute for Drugs and Medical Devices (*Bundesinstitut für Arzneimittel und Medizinprodukte*; BfArM). Only then will an app be included in the list of reimbursable digital health apps, the so-called DiGA directory. In September 2023, this directory comprised 48 admitted DiGAs [6]. DiGAs cover a wide array of the medical spectra. Popular examples are “Endo-App” (to treat endometriosis), “Kaia COPD” (for chronic obstructive pulmonary diseases), or “sinCephalea” (for the treatment of migraine).

The introduction of the “Act to Improve Healthcare Provision through Digitalisation and Innovation” represents a significant and innovative change to the German health care system [7]. The new legal framework elevates DiGAs to the ranks of medical devices [5]. Therefore, if physicians want to prescribe a DiGA, they are required to thoroughly inform themselves about which apps can help their patients, how they are to be used, and how a positive influence is expected to manifest. Patients are required and need to be able to adhere to the prescription; for example, they have to use the DiGA as stated by the physician. Thus, the physician needs to assess (implicitly or explicitly) whether the patient is likely to use the DiGA as prescribed. This does not only include adherence to, for example, training intervals (for orthopedic DiGAs) but also the general technology savviness of the patient, for example, whether the patient is able to download the app, install it on the smartphone, maintain updates, and so on. This question is specifically challenging when it comes to older users, who are often regarded as being not technology savvy.

Consequently, this innovation raises several questions for technology adoption research. Typically, adoption research concerns the individual user’s decision whether to use technology, either mandatory [8,9] or voluntary [10]. Now, a concerned third party (the physician) decides on behalf of the user whether the patient is expected to be willing and able to use an app on their smartphone voluntarily. Therefore, the physician’s assessment now includes not only the medical relevance of the DiGA but also whether the user might be able and willing to use it as prescribed. Although the latter assessment seems easy for younger people, the case is much more difficult for older patients. Potential doctor misperception raises concerns about a possible digital divide and ageism by doctors. A recent report by the WHO and the United Nations (UN) raises awareness of this issue and urges action to combat ageism, as it negatively impacts well-being and can lead to premature death and higher health care costs [11].

In the health care context, the resulting research questions are specifically relevant, as the influence of age on the adoption of mHealth apps has not yet received sufficient attention in the scientific discourse [12]. Therefore, this research addresses the complementary research questions:

1. What factors enable or hinder physicians to prescribe DiGAs?
2. What factors enable or hinder older users’ adoption of DiGAs?

## Methods

### Study Design

We developed an exploratory qualitative study design to answer the research questions and gain insights through semistructured interviews with representatives of the 2 relevant stakeholder groups: physicians and patients. The study adhered to the standards for reporting on qualitative research [13].

### Study Setting

The data for our research were gathered in Germany.

### Participants

The first qualitative study was conducted from August 2020 to June 2021. We interviewed 28 physicians (demographics are provided in Table 1) to assess how DiGAs can improve the health of their patients. Physicians were chosen as research objects because of their unique role as prescribers of DiGAs. The interviewees had different backgrounds in terms of IT affinity and previous experience with mHealth apps in general.

**Table 1:** Demographics of the physicians.

Code	Specialty	Sex	Age (years)
E1	General practitioner and cardiologist	Male	63
E2	Dermatologist	Male	67
E3	Dermatologist	Male	45
E4	Urologist	Male	48
E5	General practitioner	Female	63
E6	Dermatologist	Female	54
E7	Dermatologist	Male	41
E8	Internist and gerontologist	Male	38
E9	Orthopedist	Male	53
E10	General practitioner	Male	64
E11	Geriatric therapist	Female	58
E12	Urologist	Male	38
E13	General practitioner and pain therapist	Male	59
E14	Assistant doctor cardiology	Male	35
E15	Urologist	Male	42
E16	General practitioner	Male	68
E17	Dermatologist	Female	36
E18	General practitioner	Female	38
E19	General practitioner	Female	59
E20	General practitioner	Female	41
E21	General practitioner	Female	45
E22	General practitioner	Male	45
E23	Neurologist	Male	44
E24	General practitioner	Male	67
E25	Neurologist	Male	65
E26	Neurologist	Male	42
E27	Molecular neurologist	Female	45
E28	General practitioner	Female	37

In the second study, we interviewed patients (ie, the potential users of a DiGA). To reflect the specific issues of aging patients, we chose interview partners over 50 years of age. Evidence shows that from this age onward, chronic diseases increase significantly [14]. Therefore, this age group is likely to represent a large part of the target group for the prescription of DiGAs. Furthermore, studies suggest that there are still age disparities in attitudes toward technology and that the aging population is often less comfortable using technology [15].

We conducted 30 interviews to determine the factors that influence potential patients to adopt DiGAs or not. Demographics of the interviewees are given in Table 2.

**Table 2:** Demographics of the patients.

Code	Sex	Age (years)
P1	Male	68
P2	Female	60
P3	Female	57
P4	Female	76
P5	Female	56
P6	Female	65
P7	Male	69
P8	Female	64
P9	Male	68
P10	Female	66
P11	Male	65
P12	Male	67
P13	Female	57
P14	Female	72
P15	Female	67
P16	Male	53
P17	Female	61
P18	Female	69
P19	Female	61
P20	Male	63
P21	Male	65
P22	Female	68
P23	Female	67
P24	Female	61
P25	Female	67
P26	Female	59
P27	Male	64
P28	Female	69
P29	Female	64
P30	Female	68

### Recruitment

We recruited physicians by telephone from a community-based physician network in Germany and via social media. For the interviews with patients, we promoted our study to doctors and approached medical centers and clinics. To identify suitable interview partners (ie, *potential* patients), we presented the research project to local sports, communication, and civic groups; promoted the study on various social media platforms; and spoke to the local press. In this way, we motivated suitable candidates to contact us. The participation of all study participants, both physicians and patients, was voluntary.

### Data Collection

All interviews were semistructured and led by a list of questions and general topics that the interviewers were supposed to address. The semistructured interview guidelines are provided in Multimedia Appendix 1. As a structure and topic basis for creating the questions, the constructs from well-known technology adoption research models (unified theory of acceptance and use of technology [UTAUT] [9]) and models for the analysis of health behavior (health belief model [HBM] [16]) were considered. The questions were primarily open and allowed the interviewees to explore their experiences and views. Supported by a systematic and comprehensive interview process, the interviewers had high degrees of freedom to conduct the interview in order to gain deeper insights. The questions were adjusted correspondingly for the following interviews to gain more profound knowledge for each interview. Interviews lasted around 20-45 minutes and were conducted face-to-face or over the phone by 1 researcher (TS). The interviews were conducted in German and were recorded, transcribed, and translated into English for further analysis [17].

### Data Analysis

**Figure 1:**
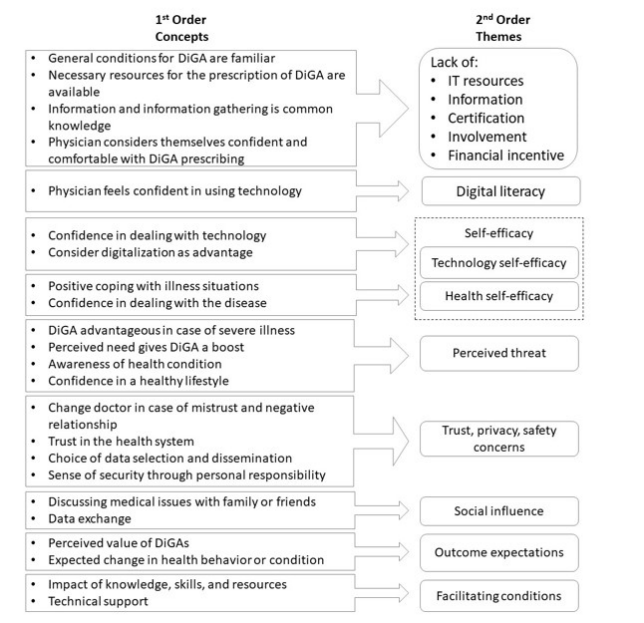
Coding scheme using the methodology by Giola et al. DiGA: Digitale Gesundheitsanwendungen (digital health application).

For the coding process, we used the NVivo10 software (QSR International). The research was conducted using an interpretative phenomenological analysis (IPA) [18]. IPA aims to examine the world perspective of the participants and, if possible, take an “insider perspective” [18] of the phenomenon under investigation. At the same time, IPA recognizes that research is a dynamic process. In parallel with the data collection, we scanned and coded the data from the first round of analysis. One researcher (TS) conducted a thematic analysis to identify patterns and themes. After analyzing the first 3 interview transcripts, 2 researchers (MH and TS) developed a coding framework using an inductive approach that allowed for identification of predominant themes. All emerging themes were cross-checked and discussed within the whole research team and developed iteratively to ensure definition and reliability. In the process, commonalities and differences between the respective perspectives were identified. This led to 9 different key themes concerning benefits and barriers, influencing factors on the intention to adopt a DiGA, and the outcome expectation from different perspectives as the main areas. Figure 1 lists the corresponding coding scheme according to Gioia et al [19]. The aim was to understand the specific properties of these areas and the influence of these factors in the context of the introduction of DiGAs.

### Ethical Considerations

This study did not require ethical approval according to the guideline of the applicable Ethics Committee of the Bavarian Universities [20], as no risks or harm was brought forward to the participants. All participants received an information and consent form explaining the requirements for participation. This included the opportunity to have the form explained to them if needed. All interviewees gave written or verbal consent before the interview started. Data collection, storage, and analysis were conducted in adherence to European Union General Data Protection Regulation (EU-GDPR). None of the participants were compensated.

## Results

### Overview

Our data demonstrate that only 4 of the interviewed physicians already prescribed DiGAs and none of the patients had used a DiGA before (Table S1 in Multimedia Appendix 1). Nevertheless, most physicians have experienced mHealth apps themselves. These were mostly used for medical information in their professional routine or privately for personal fitness and nutrition goals.

On the patient side, 20 participants had already had experience with mHealth apps or were actively using them (Table S2 in Multimedia Appendix 1). In this context, only fitness and nutrition apps were mentioned. These apps have a preventive character to help people to consciously lead a healthier and more active life. Most of the apps mentioned were those used in conjunction with smartwatches to measure activity, such as pedometers.

### The DiGA Adoption Process of Physicians and Patients

**Figure 2:**
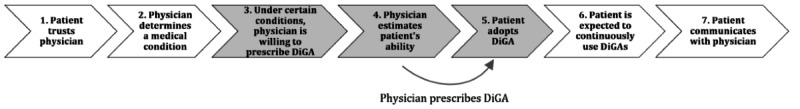
The adoption process for DiGAs by the physician and patient. DiGA: Digitale Gesundheitsanwendungen (digital health application).

The analysis indicates that for DiGAs in particular, additional steps are added to the traditional prescribing process. Because of the special role of the doctor as a gatekeeper, the doctor is the first to decide whether the patient is suitable for a DiGA. First, the familiar steps such as the patient’s trust in the doctor and the treating doctor’s determination of the patient’s medical condition represent the keystone of the process. Following these steps, when prescribing a DiGA, the next steps are the doctor’s consent to prescribe a DiGA under certain conditions and the assessment of the patient’s ability to use a DiGA. Thereafter, the doctor prescribes a DiGA, and the patient is given the opportunity to adopt it. The process ends with the expectation that the patient will continuously use the prescribed DiGA and report the results to the treating physician. This process is visualized in Figure 2, with the doctor and the patient being influenced by different factors that determine whether the DiGA is prescribed (doctor’s perspective) and accepted (patient’s perspective).

### Factors Enabling or Hindering Physicians to Prescribe DiGAs

#### Overview

Our results indicated that DiGAs are not yet widely known and used at this early stage by the physicians we interviewed. They indicated that the level of information is still insufficient and that very few DiGAs are prescribed compared with the prescription of drugs. DiGAs represent a completely new and innovative approach, so entry challenges are not considered unusual. Nevertheless, these challenges are initially barriers to prescribing DiGAs according to our participants. We were able to identify the following factors that influence physicians to prescribe a DiGA or not.

#### Lack of IT Resources

The lack of IT resources was mentioned by the interviewed doctors as a barrier to prescribing DiGAs within the framework of the DVG. Numerous efforts have been made toward digitalization. These include networking with various players in the German health system, such as doctors, hospitals, pharmacies, and health insurance companies. So far, only limited resources are available for the implementation of these plans. The doctors interviewed describe that, for example, the provision of services for an electronic health record (EHR) could help to digitalize various processes and information. They see the benefit of a DiGA for their work as low as long as the patient files and the exchange between other actors in the health care system are not fully digitized. The introduction of an EHR in Germany has failed so far because of technical challenges.

#### Lack of Information

Another reason often mentioned in connection with rejection was a lack of information. Physicians often do not know which DiGAs are available and where they could get the necessary information. As a result, physicians are often reluctant and skeptical about prescribing DiGAs (“No, I have not been educated on what DiGAs are available, how to prescribe, and how to tell if a DiGA is effective. I would not know where to look for this information” [E2, E3, E8, E12, E16, E20, E21, E25, and E28]; “I have little time to search for all the information I need to prescribe a DiGA in good conscience” [E1, E4, E8, E15, E16, E20, and E21]). They tend to be negative out of concern for malpractice and the resulting liability risk. Likewise, the physicians described the requirements for evidence of a DiGA (requirements for security, functionality, data protection, information security and quality, and positive effects on care) in the assessment procedure of a DiGA as insufficient. Along with this concern is the fact that for provisionally included DiGAs, evidence-based studies on the benefit of the app are not yet available, and the apps are therefore only provisionally included in the directory. A comprehensive explanation of the BfArM authorization process of a DiGA can counteract these problems. However, participants reported receiving little information from insurers, DiGA manufacturers, or the BfArM. Physician respondents stated that proactive communication from DiGA providers was limited and possible involvement in the development process was unknown (“These apps have often been developed without the support of physicians and are now being ‘fast-tracked’ to market—that is not exactly building trust” [E25]). This statement reflects a high level of mistrust. In contrast, one participant stated that he worked with an app development company to develop an app in his field and described that this approach was extremely helpful to reduce the information deficit, mistrust, and gain more confidence. This shows that involvement is possible, but that an exchange of information is necessary.

#### Lack of Certification

In medical circles, a lack of certification has been widely mentioned. Half of all interviewed physicians agree that DiGAs, similar to other medical products (eg, drugs and medical aids such as wheelchairs or bandages), should be provided with a known official certification to ensure more trust in the product (“I think a well-known and recognized certification in medicine could help to build confidence in DiGAs” [E2, E13, E15, E17, E26, and E28]). Certification would make it easy to recognize that the DiGAs are safe as a medical device and are also medically or technically suitable within the scope of the intended purpose stated by the manufacturer. A certification equivalent to that for medicines could be conceivable in this context.

#### Lack of Involvement

Another aspect that our interviewees criticized was the lack of involvement after the prescription of a DiGA. They described that they are little involved in the procedure after prescribing a DiGA and in many cases currently only take on the role of a “prescriber” (“I would like to be involved in the whole process from the manufacturing to the evaluation of the data with the patient” [E5]). This shows that the participating physicians apparently make a great distinction between a traditional medicine in the form of medication administration and the use of digital components. After prescribing a traditional medicine, the physician only gets feedback when the patient comes back after some time and tells them how the medicine works. But with digital options such as DiGAs, the requirement is now higher: participants demanded an adjustment of the involvement in the postprescription process. A preview of future digitization plans shows that the involvement of physicians in the digital feedback process will be considered.

#### Lack of Financial Incentives

Participants further stated that counseling for a DiGA is much more time-consuming than for medicines, but the monetary incentive is not there. As a result, we identified a lack of financial incentive. Financial pressure weighs on the physicians in this regard, which is not compensated for by health insurance companies. As a result, the incentive for prescribing (€2 [US $2.16] per prescription) and treatment support (eg, successful monitoring) is currently considered too low (“2 euros for prescribing or 7 euros for treatment support are in no way an incentive to prescribe a DiGA” [E2]).

Finally, the study also found differences in physicians’ skills, knowledge, and attitudes toward digital technology. We define this factor as digital literacy, which has 2 specific characteristics. Some of the surveyed physicians stated that they have the impression that a physician is either digitally interested and very open-minded or completely ignorant of new innovations, so that even educational conversations might fail (“Either you find it good as a physician and have dealt with it once or recommended it to your patients. Or you ignore it at first” [E6]). Along with this result, the physicians interviewed expressed the fear that they might lack knowledge, for example, when it comes to patients’ technical questions or that they would have to become a kind of “technical support” in the event of problems in this area or in the evaluation of DiGA analyses. Others, however, considered the introduction of DiGAs to be an advantage for their work and less of a hindrance or a problem.

### Factors Enabling or Hindering Older Users’ Adoption of DiGAs

#### Overview

As indicated above, a process of influencing factors leads to the adoption of DiGAs by patients. Thus, if the physician determines a medical condition, is willing to prescribe a DiGA, and considers the patient’s ability to use a DiGA to be positive, the physician will eventually prescribe a DiGA so that the patient will have the opportunity to adopt it.

#### Lack of Information

Similar to the physicians, we also identified a lack of information on the patient side. The DiGA concept is rather rarely known by the patients, and experiences were only described in 3 of the 30 interviewed participants. Nevertheless, from the patient perspective, DiGAs are recognized as an innovative and profitable treatment option that can be carried out independently of the time and place of the doctor’s visit (“I want to have the flexibility to do my therapy when it suits me” [P1, P6, P9, P12, P18, P26, P29, and P30]). Patients described DiGAs as a helpful “bridge” and a refresher or repetition of therapy content, especially for patients waiting for an appointment with a specialist (eg, psychotherapist). Some patients pointed to a long history of illness and low chances of success of conventional therapies and considered DiGAs as another treatment option. The interviews thus confirmed that the population’s willingness for DiGAs is high. However, many patients lack further information (“My physician or my health insurance company haven’t informed me about it yet—how should I know?” [P2, P3, P12, P16, P19, P22, and P29]).

The factors influencing the adoption of DiGAs are discussed in more detail below. In this context, we are oriented toward the most well-known models in adoption research: the UTAUT [9], a model from information systems that measures the acceptance of a technology by users to gain access to individual user behavior, and the HBM [16], a theoretical model from health psychology that analyzes and predicts health-related behavior.

#### Self-Efficacy

Self-efficacy refers to a sense of control over one’s environment and behavior. Participants considered self-efficacy important but also taken for granted. A distinction was made between technology self-efficacy about the DiGA and health self-efficacy with a health aspect. We define technology self-efficacy as the patient’s belief in their competence to use the DiGA. Health self-efficacy here describes the patient being confident in managing their health. Participants expressed confidence in their technology self-efficacy. For all participants, everyday use of smartphones and various apps was normal and regular. Some indicated that they still prefer paper calendars or dislike apps for health prevention but saw digital documentation and treatment support as an advantage in case of possible illness (“I’m more used to pen and paper, but if you get used to it, it certainly has its advantages” [P1, P4, P12, P14, P21, and P29]). In terms of health self-efficacy, participants were mostly self-confident. No participant stated that they did not want to deal with illnesses or would rather not know how healthy or ill their body was. Most participants seemed to have a high level of health literacy and wanted to actively deal with their health (“Yes, I would rather be the person who would then say, I would like to know this to know my enemy and then fight against it” [P19]). Participants indicated that a personal sense of control facilitates health behavior change (“Then an app like that would be great because you can calm down a bit more and have more security for yourself” [P10]). Participants confirmed that they focus on the opportunities rather than the obstacles (“Yes, I usually don’t go right away, but after a short scare I always face all the problems, so I’m more for problem solving rather than suppression” [P3]).

#### Perceived Threat

Perceived threat was mentioned as a crucial central factor for the use of a DiGA in the context of an impending or existing chronic disease. According to the HBM, perceived threat stems from beliefs about perceived susceptibility to disease and the perceived severity of disease consequences [16]. Susceptibility here refers to a person’s risk of contracting a disease. Severity refers not only to the medical consequences but also to the potential impact of an illness on a person’s daily life, family life, and social relationships. Participants indicated that a threat has a strong influence on health behavior. They explained that if they were seriously ill, they would use any means to support treatment, both digital and analogue (“So, I would say if I got sick now, I would be very interested in a DiGA already” [P29]; “Yes, when I get sick, I try everything possible to get better, whether digital or not” [P3]). Nevertheless, participants indicated that they generally felt very body and health conscious (“I eat healthy and exercise as much as I can, that’s part of my everyday life” [P9, P11, P15, P16, P21, P23, P26, and P29]). Only 1 respondent indicated that they felt an increased fear of possible illness. All participants indicated that they were primarily concerned with their health and well-being and that preventive measures were a natural part of their lives (“I prefer to focus positively on my health instead of worrying daily about illness and negative health” [P23, P29, and P31]).

#### Trust in the Physician, Attitudes Toward Privacy, and Safety Concerns

Partly different from classical adoption research, trust in the physician, attitudes toward privacy, and safety concerns were mentioned as key characteristics. Due to the medical field, the existing models require expansion. Trust does not describe a direct influencing factor but rather a precondition. For the participants, a deep trust relationship with their physician was crucial to consult a physician in case of a health problem and to receive good medical treatment. If this precondition is not given, an exchange about digital treatment options does not take place (“If I feel that I cannot trust him, then I would change” [P1, P7, P10, P13, and P22]; “The most important thing is the trust relationship. If I don’t trust my physician, I change physicians” [P27]). Subordinated are the characteristics of privacy and security. All participants considered it important to be able to set privacy settings themselves. The vast majority said that they did not particularly care about the content of privacy settings, but wanted to decide for themselves who could access which health data (“I would like to know who knows what about me” [P6, P7, P8, P15, and P26]; “There should be settings options. I don’t want to transmit everyday occurrences” [P9, P10, P11, P13, and P27]). Based on the accreditation of a DiGA as a medical device and the assessment by the BfArM, they have no concerns about the safety of DiGAs and feel confident in using DiGAs (“I trust that our federal system is highly secure” [P11]). On the basis of these correlations, we categorized the 3 concepts together. We rank the trust factor as the most important, as the privacy and security factors can be mitigated by a high level of trust.

#### Social Influence

The concept of social influence reflects the effect of environmental factors, for example, the opinion of friends and family, and is a significant factor in traditional adoption research [21]. However, in this study, we could not find any relevant results. Here, the medical context seems to have an important role. Most participants indicated that they did not discuss their health behaviors, personal diagnosis, or treatment plans with their social circle (“I don’t want to share all health data, including the fact that I use a health app, with other people” [P9]). We suspect that social influence may be a factor that is difficult to capture due to the sensitive nature of the data.

#### Outcome Expectations

Outcome expectations are defined as the expected consequences of a certain health behavior, which can be negative or positive [22], that is, what does the patient expect from using a DiGA. Participants stated that they would be able to reduce the frequency of visits to the physician, thus saving travel and time; that an existing illness would be better monitored by the physician; that they would feel safe and well cared for as a result; and that they would receive health-promoting treatment in the form of the DiGA. However, these expectations were tied to the continued use of features of the DiGA. Participants agreed that a DiGA must be simple to use, regardless of age. Many participants expect the app to provide more detailed information about the disease, symptoms, medication, and contraindications. However, this information needs to be understandable, meaningful, and informative for every patient, regardless of age, education level, and professional background (“It should be easy for me, and I should be able to understand and comprehend it. The ease of use.” [P1, P5, P16, P19, and P21]).

#### Facilitating Conditions

Eventually, these expectations also cross over to the facilitating conditions, which includes the impact of the patient’s knowledge, skills, and resources. Here, the participants considered technical support to be particularly important. This was not because they felt unsure about using the technology but rather because it was a new type of health intervention. Some participants considered the physician to be an appropriate point of contact when difficulties arose in a few cases. Other participants stated that if they had difficulties or questions, they would ask their family and friends for help, as they do with other technical matters (“I think it would be good to have a number that I can call and that can help me” [P2]; “Well, I trust my son, he knows me anyway. And he would also know the diseases I have” [P18]). In summary, this study distinguishes between technical infrastructure and health support, with the health aspect (eg, knowledge and understanding of the diseases and their treatment) being more important here.

### Post Hoc Analysis: Physician Assessment of the Patient’s Ability to Use a DiGA

During our interviews with the physicians, we discovered a phenomenon that we did not initially anticipate and that has not yet been described in the relevant technology adoption literature: the pre–user adoption decision of another instance, whether the user will be able and willing to voluntary use a technology. Typically, in technology adoption research analyses, this means whether a user is willing to use a technology in an either voluntary or mandatory environment. In any case, the decision remains with the user.

Now we see a new phenomenon: the assessment of one instance (physician), whether a subsequent instance (patient) would be able to use the DiGA. Only if the assessment is positive would the physician offer the DiGA to the patient, which will then trigger the traditional adoption questions and corresponding behavior of the user, as described in the well-researched technology adoption models such as the technology acceptance model, UTAUT, and HBM [9,16,23].

There are no guidelines under which circumstances a patient should be assumed to be able to use a DiGA. Thus, each physician needs to do this assessment individually. If they conclude that the patient will likely not be able to install, maintain, and use the app as foreseen by its developers, there is no point in prescribing the DiGA. As there are no objective guidelines, the assessment is either done explicitly, by asking the patient, or implicitly, by assuming what the patient is capable of.

Interestingly, the physicians interviewed were very consistent regarding the assessment of digital literacy of their patients. They indicated that prioritization of certain patient groups is facilitated by anticipating the digital literacy of their patients. Unfortunately, this often leads to a negative bias toward older users; the physicians described the typical DiGA user as a young and tech-savvy patient (“I would not consider my older patients for the use of DiGAs” [E1, E2, E4, E7, E8, E11, E12, E15, E16, E17, E20, E21, E22, and E28]; “There are certainly exceptions, but most of my older patients are totally overwhelmed with a tablet or a smartphone, because the interest would not even be there” [E11]). Consistently, physicians expressed that they would not even consider an older patient as a DiGA user.

As mentioned before, these findings arose from the data and were not anticipated before. Thus, both issues, the second-order technology adoption process and the (possible) systematic disadvantage of older users, need deeper investigation in further research.

## Discussion

### Overview

In this research, we identified the salient factors that were either beneficial or hindering the adoption of DiGAs from the physician and the patient perspective. Furthermore, the results of our study suggest that the adoption process for a DiGA does not only depend on patient behavior but also on the physician’s behavior.

Most informants have a positive attitude toward the digitalization in general. Nevertheless, physicians’ demands on DiGAs are high, and their perceptions can be affected by a lack of facilitating conditions, trust, and digital competence. Certain influencing factors for the adoption of DiGAs by patients are consistent with the literature on established adoption research [9,24-28].

### Principal Implications

Our study contributes to the field by investigating factors influencing the adoption of DiGAs to inform future research and guide strategies and efforts for this user group. DiGAs represent a wide range of assistive apps that aim to support disease behaviors, manage various health conditions, and maintain the well-being of those with chronic diseases. There are very few empirical studies addressing the factors influencing users’ adoption of DiGAs [7,29,30]; hence, there is limited knowledge and guidance from the existing literature.

First, it is important to demonstrate that existing technology acceptance models reach their limits when used in the context of DiGAs. In contrast to Davis [23] and Venkatesh et al [9], our interviews with physicians and potential patients led to the assumption that, in addition to usefulness and ease of use, there are more constructs that play a significant role. We found that technology and health aspects such as technology- and health-related self-efficacy, trust, and a trustful doctor-patient relationship play a major role in the intention to use DiGAs. So far, these aspects have rarely been brought together. A study by Uncovska et al [30] confirms this finding. However, we note that there are few studies on the adoption process of DiGA. Other studies regarding mHealth app adoption have highlighted that health consciousness of individuals is a factor that directly influences both the intention to use mHealth apps and the actual use behavior [31,32]. Public trust in the health care system [33] and a strong doctor-patient relationship can empower patients to contribute to treatment decision-making [34].

Second, we found that our interviewees on the patient side distinguished between technology-related and health-related self-efficacy. The consideration of a health component is not integrated into traditional technology adoption research. To date, it has not seemed necessary to consider the health domain in adopting general technologies. However, this is an important difference in the adoption of DiGAs. With DiGAs, the focus is on the health aspect for both the physician and the patient. Patients who may have low self-efficacy with technology do not simultaneously have to have low self-efficacy with their health. Older people, in particular, may have very sophisticated health self-efficacy while lacking technology self-efficacy [35-38]. Distinguishing these forms of self-efficacy provides a more detailed explanation of adoption behavior, increasing our understanding in the context of DiGAs. Personal beliefs, such as outcome expectations and self-efficacy expectations, are among the most critical variables in terms of intention formation and bridging the gap between intention and behavior, according to existing literature [39-41]. Nevertheless, a division into different areas of self-efficacy has not yet been made in information systems research but also in research on health adoption. As technology and health self-efficacy positively impact the adoption of DiGAs, we believe that it is important to consider both factors.

Third, related to the previous aspect, is the construct of perceived threat. Previous research shows that people are concerned about adopting technology in different areas, such as privacy, effort, or performance [7,42,43]. However, when using DiGAs, health is firmly in focus from a medical perspective. Therefore, the perceived threat of diseases and the need to use a DiGA strongly influenced the adoption of a DiGA. In our interviews, this construct was strongly emphasized, and we suspect moderation effects on other constructs, such as technology and health self-efficacy. A high perceived threat can increase the influence of the perceived health self-efficacy on adopting a DiGA because a threat can be better assessed by someone with high health self-efficacy and is, therefore, more likely to act. As a result, it is also possible that DiGAs will have a higher use rate, especially for hazardous diseases. A recent study by Pourhaji et al [44] investigated the perceived threat and stress response to the COVID-19 pandemic and found that the Iranian population’s health behavior was influenced by the perceived severity and susceptibility of the infection, which meant that preventive interventions were more likely to be accepted. Further studies related to COVID-19 found that risk severity also tends to increase with age, but the perception of susceptibility to contracting COVID-19 decreases [45-47]. Thus, risk perception does not seem to increase with age, but vulnerability and severity show opposite patterns [48]. The HBM postulates that individual beliefs about risk can be influenced by various factors such as sociodemographic and sociopsychological variables as well as knowledge, experience, and awareness [16]. However, patient awareness can also become an important issue, as these patients may not perceive a threat and, therefore, not adopt a DiGA.

Fourth, in addition to the patient, this study involves another important stakeholder, the physician. This stakeholder is not considered in the technology adoption models as they do not provide a specific gatekeeper for the technology or basically consider different stakeholders. But in the case of DiGA adoption by the patient, the first step requires the physician’s adoption of a DiGA. This observation has also been noted in previous studies [7,49-52]. Subsequently, the physician’s positive assessment of the patient’s competence to perform a certain behavior is one of the essential conditions for a patient to consistently perform a health intervention. This result relates to previous research without reference to DiGAs as well as with reference to DiGAs [7,53]. Similarly, this view can be developed in a negative direction when doctors decide that the patient is not capable of adopting and using a DiGA, which could be justified by digital ageism. Ageism is a societal bias conceptualized as (1) prejudicial attitudes toward older adults, (2) discriminatory practices toward older adults, or (3) institutionalized policies and social practices that promote these attitudes [54]. Ball et al [55] show that both the development and use of technology have excluded older adults, resulting in a “physical-digital divide,” which exists when a group feels excluded because they are unable to engage with the technologies used around them. Some studies suggest that ageism is widespread in the health care system [56-58]. For example, Walter et al [59] showed that physicians promote less preventive care for older patients. Chu et al [60] emphasized that the exclusion of older people from technology development leads to a broader cycle of inequity and ageist social attitudes, widening the digital divide. In contrast, we noticed that the trust factor impacts a patient’s health behavior, which is in line with Wildenbos et al [61]. Beyond this, we also found that the physician’s trust in the DiGA is equally important for their prescription of a DiGA. A physician needs a strong relationship of trust with the patient to convince the patient of the treatment methods. By motivating their patients to adopt a DiGA and use it to support their therapy, physicians focus on their social influence on the patient [7,62]. We provide justification for the incorporation of the physician as an important influence on adoption behavior in this context. After all, DiGAs live and die with physicians’ willingness to prescribe to their patients and influence them to understand the technology’s necessity. We argue for an adoption model that does not only incorporate a human-technology interaction but also a human-human-technology interaction.

### Implication for Practice

The results of our study demonstrated that there is insufficient information available and published for both physicians and patients, as well as a lack of comprehensive technical support. Some statements (eg, the involvement of medical professionals in the development process of a DiGA, DiGA list unknown, and lack of evidence-based sources) confirm the knowledge deficit. It is important for health policy makers and public authorities such as the Ministry of Health, the Medical Association, and insurers to address these issues. Extensive information and source references are needed to take into account the needs of physicians and to enable DiGAs to get started more effectively. Including the consideration of conflicting goals in technology development from the beginning seems necessary. Codevelopment can improve app use and effectiveness in the long term by using a user-centered design to develop DiGAs that are effective in chronic disease self-management [63,64]. Likewise, we demonstrated that physicians distinguish clearly between digital and traditional treatment options and have significantly higher expectations of digital resources. Despite a previously excluded position of the physician in the development and introduction process of medicines, the physician now anticipates more involvement in digital developments. In order to counteract the feeling of exclusion and disconnection from the supposedly nonmedically focused DiGA development, appropriate education seems to be required. One way to achieve this is through promotion within public networks, local authorities, and medical associations.

Insufficient participation in the follow-up to a prescription was also a concern for medical practitioners. In this context, it is important to fully communicate to physicians the many opportunities DiGAs offer. DiGAs have higher potential than other treatment interventions such as medicines to maintain a meaningful exchange of information and stay in touch with the patient even after prescription. We found that physicians feel a loss of control when the DiGA is prescribed and then used by the patient. A high degree of self-management is demanded of the patient, leaving the physician feeling incapable of action. Digital monitoring with the help of a DiGA also results in an advantage after the prescription compared with the conventional prescription of medical devices and medicines. Furthermore, negative attitudes and lack of digital competence among physicians are major barriers to physician prescription of DiGAs and, thus, patient adoption. At the same time, the lack of facilitating conditions and the high demands regarding the introduction of various digital changes (eg, EHR) exert excessive pressure on physicians. In this case, further information and education of physicians would be useful. In addition, a trial period of the DiGA can demonstrate digital connectivity to the physician.

### Limitations and Future Research

Because of the exploratory character of the research design, our findings naturally lack generalizability and should be regarded as a first starting point on the investigation of a new phenomenon. As DiGAs are a new option for physicians, several interview partners have not really experienced them yet. We found that some participants, especially those who had not yet heard of DiGAs, found it difficult to properly understand the use and benefits of DiGAs.

Another issue corresponding to the novelty of the phenomenon is that informants’ perceptions change quickly. Therefore, our findings reflect the perceptions of interview partners in the early phase of introductions of DiGAs into the market. It is likely that some of the issues raised will not be present in a couple of years, when DiGAs are more common to the market and perceived as natural to prescribe as all other medicines today.

This poses interesting questions for further research. It would be interesting to conduct longitudinal studies to gain a better understanding about the diffusion of such innovations in the medical space from the legislative setting into the physicians’ toolkit and finally to the patients. This could generate valuable insights for future management of digital innovations in the medical area. In close conjunction to these questions, a cross-national comparison could generate advice for policy to smoothen the introduction phases of digital medical innovations in new other countries.

Finally, the study of the second-order adoption mechanisms—highlighted earlier in the document—could lead to interesting theoretical insights and valuable advice for practitioners to enhance the prescription and adoption of DiGAs and comparable digital innovations.

### Conclusions

DiGAs provide an opportunity to support people with severe (often chronic) diseases, to live independently with greater confidence and understanding of their condition, better symptom management, and ultimately enhanced quality of life. Our study provides deep insights into the needs and circumstantial evidence that enables a better understanding of the perspectives and preferences for adopting DiGAs by physicians and potential patients. We found that there is a considerable lack of information on both physicians’ and patients’ sides, resulting in poor trust and digital competence. Furthermore, we identified several factors influencing the adoption of DiGAs, which led to a new understanding of adoption research concerning digital health technologies.

## Appendix

Multimedia Appendix 1 Interview guidelines and descriptive results.

## Abbreviations

BfArM: Bundesinstitut für Arzneimittel und Medizinprodukte (Federal Institute for Drugs and Medical Devices)
DiGA: Digitale Gesundheitsanwendungen (digital health applications)
DiGAV: Digitale-Gesundheitsanwendungen-Verordnung (Digital Health Applications Ordinance)
DVG: Digitale-Versorgung-Gesetz (Digital Healthcare Act)
EHR: electronic health record
EU-GDPR: European Union General Data Protection Regulation
HBM: health belief model
IPA: interpretative phenomenological analysis
mHealth: mobile health
UN: United Nations
UTAUT: unified theory of acceptance and use of technology
WHO: World Health Organization
