# Supplementary material for: Evidence of How Physicians and Their Patients Adopt mHealth Apps in Germany: Exploratory Qualitative Study
Source: JMIR Mhealth Uhealth. 2024 Jan 17;12:e48345. doi: 10.2196/48345 (PMC10831587; doi:10.2196/48345)
Supplement: Multimedia Appendix 1 [file mhealth_v12i1e48345_app1.docx]

Multimedia Appendix 1: Interview Guidelines

### Semi structured interview guide—physician

1. Introduction
   1. Warm up and ask for time frame.
   2. Objective of the interview/study
2. Differentiation between DiGA and health apps
3. Find factors in favor and against the use of DiGA.
4. Attitude of doctors towards DiGA.
5. Will patients use DiGA (in the long term)?
6. Introduction of interviewers (background and research focus).
7. Question about audio recording, reference to anonymity.
8. Start recording.
9. Interviewee (age, position/role - focus, since when, type of practice?)

IT Competence:

1. General IT competence?
2. Experience with smartphone apps?
3. Do you use health apps privately? (Differentiation from DiGA)

Object of investigation DiGA

1. Which DiGA do you know?
2. How are you informed about DiGA? Suggestions for improvement?
3. How well do you feel informed about the use of a DiGA? (Only info flyer?)
4. Do you have the opportunity to test DiGAs yourself before prescribing?
5. Feedback to the information points? E.g., possibilities for a DiGA where none currently exists?

Attitude to the subject of the study:

1. Have you ever prescribed a DiGA?
2. Reasons for prescribing/not prescribing?

**Contra Case:**

1. What reasons? (open question)
2. Possible points
   1. Data protection, concerns if data is held by third party providers?
   2. IT security problems
   3. No benefit for the patient
   4. Lack of IT competence for the patient
   5. Too complicated to use for the patient (process after prescription)
   6. Lack of information for the doctor
   7. Lack of IT competence of the doctor
   8. No feedback on use to the doctor
   9. Confrontation with chronic disease not wanted.
3. Do you know colleagues (other doctors) who prescribe DiGAs or also reject them?
4. Then continue with optional questions and the conclusion of the Interview (No. 35-38)

Patient assessment

1. How IT-savvy do you consider your patients to be?
2. Do your patients’ own smartphones or tablets?
3. To which patients do you suggest a DiGA?
4. To which patients do you not suggest a DiGA (although it would be possible)?
5. Have you ever been approached independently by patients about a DiGA?

Further process

1. What is the process for the patient with the prescription for a DiGA? What happens after the prescription is issued?
2. Have patients ever contacted you with questions about the DiGA? Could you help them?

Health and adherence

1. Ensuring the use of the DiGA? Do patients adhere to what you are told?
2. Feedback from the DiGA to the doctor? Would this be important? How should this be done?
3. Are you aware of DiGAs that also serve prevention?

Technology and social influences

1. Privacy or IT security issues with DiGAs?
2. Concerns if data is held by third party providers?
3. Do you know people who regularly use health apps?
4. Do you know colleagues (other doctors) who prescribe or refuse DiGAs?

Conclusion of the Interview

1. Optional: Are there patients who are skeptical about your diagnoses or treatment plans? For example, who suggest alternative treatments (keyword: Dr. Google, but also DiGA).
2. Optional: Do you have experience with doctor ratings (Jameda or Google)? What role do these ratings play for your practice?
3. Stop recording.
4. Thank you, instructions on how to proceed.

### Semi structured interview guide—older adult

1. Demographic data:
2. Gender?
3. Age?
4. Professional education?

IT competence:

1. Please describe your experience with smartphone apps.
2. What do you understand by a health app?

General:

1. Which prescription apps do you know?
2. What is your previous experience with prescription health apps in particular?
3. What factors can positively influence you to use a health app?
4. What factors can negatively influence you to use a health app?
5. What consequences or outcomes do you see in using a health app?
6. How would you influence these consequences in your use?
7. What risks do you perceive regarding the use of a health app?

Perceived Threat:

1. How great is your concern about becoming chronically ill?
2. How high do you consider yourself to be at risk of developing a chronic illness?

Health Self Efficacy:

1. How important is it for you to actively deal with your health?
2. What preventive / repressive measures can you imagine implementing?
3. What would be obstacles to deal with health and/or illness?
4. How confident are you in dealing with a (chronic) illness?

Health Effort Expectancy:

1. How much effort do you think it would take to actively work on your health?
2. Do you see challenges in documenting your feelings (your disease progression) in an app?
3. How might documenting sensations/disease progression influence your health behavior?

Security Concern:

1. How do you rate the security of your data in a health app?

Privacy Concerns:

1. How do you rate the privacy possibilities of your data in a health app?
2. What concerns do you have about privacy settings?

Trust:

1. Please describe the trust relationship you have with your treating doctor (GP or specialist).
2. How important is it to you to have a good relationship of trust with your doctor?

Technology Effort Expectancy:

1. What are your expectations regarding the usability of a health app?
2. How much effort do you think it would take to use a health app regularly?

Social Influence:

1. Do you know people who regularly use health apps (prescribed by the doctor or not prescribed by the doctor)?
2. Do you feel influenced by your social environment to use a health app? Or can the social environment influence you to use a health app?

Performance Expectancy:

1. How important is it to you to regularly log your health status?
2. What encourages or discourages you to log your health data?

Technology Self Efficacy:

1. How confident do they feel in using a smartphone or health app?
2. Do you often need help when using smartphone apps or health apps?

Facilitating Conditions.

1. How could you get support if you have questions or don't understand about health apps?

Cues to Action:

1. Do you find the doctor's treatment plans comprehensible, or do you follow them (or not) just because you are told to?"
2. Are you able to follow the treatment plan, are you taking your medication as advised? If no, what do you think is the reason for this?
3. What factors influence you in following the treatment plan?

Conclusion of Interview:

1. In general, how important do you think it is to keep a record of your health data?
2. How confident are you that you would use a health app regularly?

### Descriptive Results

Table S1. Descriptive results physicians.

| **No.** | **Gen-der** | **Age** | **Experience mHealth apps** | **Pres-cription DiGA** | **No.** | **Gen-der** | **Age** | **Experience mHealth apps** | **Pres-cription DiGA** |
| --- | --- | --- | --- | --- | --- | --- | --- | --- | --- |
| **E1** | M | 63 | **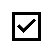** | **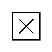** | **E15** | M | 42 | **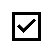** | **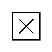** |
| **E2** | M | 67 | **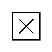** | **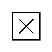** | **E16** | M | 68 | **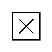** | **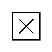** |
| **E3** | M | 45 | **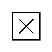** | **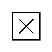** | **E17** | F | 36 | **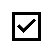** | **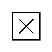** |
| **E4** | M | 48 | **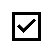** | **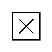** | **E18** | F | 38 | **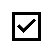** | **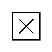** |
| **E5** | F | 63 | **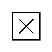** | **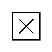** | **E19** | F | 59 | **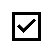** | **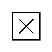** |
| **E6** | F | 54 | **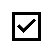** | **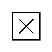** | **E20** | F | 41 | **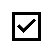** | **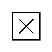** |
| **E7** | M | 41 | **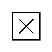** | **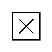** | **E21** | F | 45 | **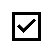** | **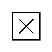** |
| **E8** | M | 38 | **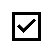** | **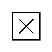** | **E22** | M | 45 | **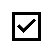** | **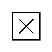** |
| **E9** | M | 53 | **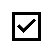** | **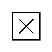** | **E23** | M | 44 | **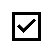** | **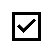** |
| **E10** | M | 64 | **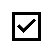** | **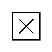** | **E24** | M | 67 | **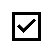** | **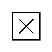** |
| **E11** | F | 58 | **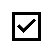** | **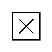** | **E25** | M | 65 | **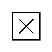** | **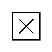** |
| **E12** | M | 38 | **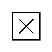** | **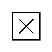** | **E26** | M | 42 | **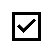** | **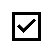** |
| **E13** | M | 59 | **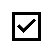** | **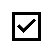** | **E27** | F | 45 | **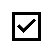** | **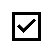** |
| **E14** | M | 35 | **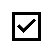** | **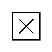** | **E28** | F | 37 | **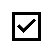** | **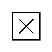** |

Table S2. Descriptive results patients.

| **No.** | **Gen-der** | **Age** | **Experience mHealth apps** | **Use of DiGAs** | **No.** | **Gen-der** | **Age** | **Experience mHealth apps** | **Use of DiGAs** | **No.** | **Gen-der** | **Age** | **Experience mHealth apps** | **Use of DiGAs** |
| --- | --- | --- | --- | --- | --- | --- | --- | --- | --- | --- | --- | --- | --- | --- |
| **P1** | M | 68 | **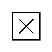** | **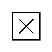** | **P11** | M | 65 | **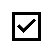** | **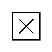** | **P21** | M | 65 | **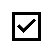** | **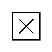** |
| **P2** | F | 60 | **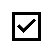** | **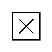** | **P12** | M | 67 | **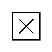** | **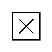** | **P22** | F | 68 | **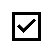** | **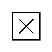** |
| **P3** | F | 57 | **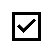** | **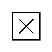** | **P13** | F | 57 | **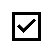** | **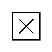** | **P23** | F | 67 | **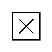** | **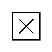** |
| **P4** | F | 76 | **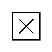** | **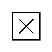** | **P14** | F | 72 | **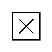** | **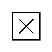** | **P24** | F | 61 | **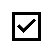** | **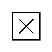** |
| **P5** | F | 56 | **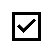** | **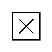** | **P15** | F | 67 | **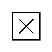** | **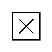** | **P25** | F | 67 | **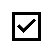** | **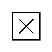** |
| **P6** | F | 65 | **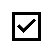** | **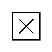** | **P16** | M | 53 | **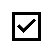** | **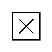** | **P26** | F | 59 | **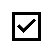** | **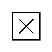** |
| **P7** | M | 69 | **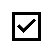** | **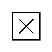** | **P17** | F | 61 | **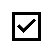** | **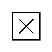** | **P27** | M | 64 | **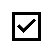** | **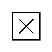** |
| **P8** | F | 64 | **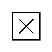** | **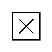** | **P18** | F | 69 |  |  | **P28** | F | 69 |  |  |
| **P9** | M | 68 |  |  | **P19** | F | 61 |  |  | **P29** | F | 64 |  |  |
| **P10** | F | 66 |  |  | **P20** | M | 63 |  |  | **P30** | F | 68 |  |  |
